# HLM Staging and Treatment Benefit of SGLT2i Across Heart Failure Severity

**DOI:** 10.3390/jcdd13080361

**Published:** 2026-08-01

**Authors:** Andrea D’Amato, Federico Ferranti, Claudia Cestiè, Alberto Casasola, Silvia Prosperi, Vincenzo Myftari, Rosanna Germanò, Camilla Segato, Giovanna Manzi, Domenico Filomena, Lucia Ilaria Birtolo, Gaetano Ruocco, Francesco Ciciarello, Alberto Palazzuoli, Massimo Mancone, Paul J. Mather, Paul N. Casale, Roberto Badagliacca, Francesco Fedele, Carmine Dario Vizza, Paolo Severino

**Affiliations:** 1 Department of Clinical, Internal, Anesthesiology and Cardiovascular Sciences, Sapienza University of Rome, Viale del Policlinico, 155, 00161 Rome, Italy; damatoandrea92@gmail.com (A.D.); federico.ferranti96@gmail.com (F.F.); claudia.cestie@gmail.com (C.C.); casasola.1977983@studenti.uniroma1.it (A.C.); silviapro@outlook.it (S.P.); vincenzo.myftari@gmail.com (V.M.); rosanna.germano@gmail.com (R.G.); segato.1841469@studenti.uniroma1.it (C.S.); giovanna.manzi@uniroma1.it (G.M.); domenico.filomena@uniroma1.it (D.F.); lucia.birtolo@uniroma1.it (L.I.B.); francesco.ciciarello@uniroma1.it (F.C.); massimo.mancone@uniroma1.it (M.M.); roberto.badagliacca@uniroma1.it (R.B.); francesco.fedele@uniroma1.it (F.F.); dario.vizza@uniroma1.it (C.D.V.); 2 Cardiology Unit “I. Veris Delli Ponti Hospital”, ASL Lecce, 73020 Scorrano, Italy; gaetanomruocco@gmail.com; 3 Cardiovascular Diseases Unit, Cardio Thoracic and Vascular Department, Le Scotte Hospital Siena, University of Siena, 53100 Siena, Italy; palazzuoli2@unisi.it; 4 Division of Cardiovascular Medicine, University of Pennsylvania, Philadelphia, PA 19104, USA; paul.mather@pennmedicine.upenn.edu; 5 Department of Cardiology and Population Health Sciences, Weill Cornell Medical College, New York, NY 10021, USA; pnc9003@nyp.org; 6 National Institute for Cardiovascular Research, 40126 Bologna, Italy

**Keywords:** heart failure, HLM, SGLT2i, prognosis, pathophysiology, pharmacology

## Abstract

Heart failure (HF) is a complex systemic syndrome for which the HLM staging system has been proposed to better capture disease complexity. This study investigated the prognostic role of HLM staging according to SGLT2i status and the impact of in-hospital SGLT2i therapy across different stages of HF severity. In this prospective, multicenter, observational study, consecutive patients hospitalized for HF between November 2022 and August 2025 were enrolled and stratified according to HLM stage and according to SGLT2i status at discharge. The primary endpoint was the composite of cardiovascular (CV) death or HF hospitalization at 6-month follow-up. A total of 711 patients were included. The primary endpoint occurred in 82 patients with a significant increase across HLM stages (*p* < 0.001). Increasing HLM stage was associated with higher risk of adverse outcomes (HR = 1.40; 95% CI 1.02–1.90; *p* = 0.03), regardless of SGLT2i status. SGLT2i therapy initiation within the index hospitalization was independently associated with a lower risk of the composite endpoint (HR = 0.59; 95% CI 0.38–0.92; *p* = 0.02) across the HLM severity stages. In this cohort of HF patients, the HLM staging system provided potential clinically meaningful risk stratification irrespective of SGLT2i therapy, and SGLT2i therapy was associated with improved outcomes irrespective of HLM severity. These observations suggest a potential role for integrated pathophysiological staging in risk assessment and therapeutic evaluation.

## 1. Introduction

Heart failure (HF) is a complex and multifaceted clinical syndrome with a high social and economic burden worldwide, as well as high mortality rate [1]. HF is not a condition confined solely to the heart, because it is a progressive disease leading to multiorgan involvement, which has an important prognostic weight. Therefore, there is an increasing interest in holistic management of these patients, moving beyond a cardiocentric approach. This new perspective can be appreciated in the most recent guidelines [1] of the European Society of Cardiology (ESC) on the management of patients with HF, in which substantial emphasis has been placed on the treatment of concomitant comorbidities such as anemia, chronic kidney disease (CKD), diabetes mellitus type II (T2DM) and obesity. Therapies targeting these multiple comorbidities have been recommended, demonstrating improvements in prognosis and/or quality of life [1]. In the current guidelines, angiotensin-converting enzyme inhibitors (ACEis), angiotensin receptor–neprilysin inhibitors (ARNIs), beta-blockers (BBs), mineralocorticoid receptor antagonists (MRAs), and sodium–glucose cotransporter 2 inhibitors (SGLT2is) are indicated to reduce the mortality and hospitalization. Notably, the latter are recommended as Class IA therapy for HF irrespective of left ventricular ejection fraction (LVEF). They also exert pleiotropic effects, acting not only as antidiabetic agents but also showing multisystemic effects [1].

This perspective highlights the multiorgan involvement in HF, recognizing it as a progressive and fatal syndrome. For this reason, and for these patients’ complexity, simple parameters such as LVEF or New York Heart Association (NYHA) class cannot accurately stratify HF patients [2]. The wide multiorgan involvement in this syndrome supports a parallel with cancer and may allow the conceptualization of HF as a progressively spreading disease [3]. As in oncology, the development of a risk score enabling patient stratification in the field of HF may represent an important tool for clinicians. In this context, further pursuing the parallel with malignancies (commonly classified according to the TNM staging system), an analogous score, termed the “HLM”, has been proposed for the stratification of patients with HF [3]. Within this model, the letter “H” refers to the heart, analogous to the letter “T” in oncology, which denotes the primary tumor. The letter “L” represents the lungs, which may be conceptually regarded as the “lymph nodes of the heart,” corresponding to the letter “N” in the TNM system. Finally, the letter “M” reflects multiorgan involvement, analogous to the presence of metastases in malignant disease [3]. As in oncology, where the clinical efficacy of a given therapy varies according to the TNM class, in patients with HF, the efficacy of pharmacological therapy may also vary depending on disease severity and the extent of multiorgan involvement. In the context of an increasingly personalized approach to HF management, better understanding how to tailor SGLT2i therapy according to disease severity may be clinically relevant. Although SGLT2is have shown consistent pleiotropic benefits across the LVEF spectrum, evaluating their effect according to HLM severity stages may help clarify their positioning within pathophysiological trajectory of HF, offering a different perspective compared to current approach.

Therefore, the aim of the present study was to investigate the interaction between HF severity, assessed through the HLM staging system, and SGLT2is, examining the clinical benefit of this drug class among the different HF severity classes as well as the prognostic power of HLM scoring system according to SGLT2i status.

## 2. Materials and Methods

The present study was an observational, prospective, multicenter study enrolling patients with a diagnosis of HF who have been consecutively admitted to three hospital centers: (i) the Department of Clinical, Internal, Anesthesiology and Cardiovascular Sciences at Policlinico Umberto I, Sapienza University of Rome; (ii) the Department of Cardiovascular Disease, Le Scotte Hospital, University of Siena and (iii) the Division of Cardiovascular Medicine, University of Pennsylvania, Philadelphia, PA, USA. Inclusion criteria were the following: (i) written, signed and dated informed consent; (ii) age above 18 years; and (iii) diagnosis of HF according to the current guidelines [1]. Exclusion criteria were the following: (i) any condition limiting life expectancy to less than one year; (ii) end-stage kidney failure and/or dialysis; (iii) planned or history of heart transplantation and ventricular assist device (VAD); (iv) pregnancy or nursing; (v) non-compliance with the study protocol; (vi) absence of relevant data; (vii) prior SGLT2i therapy.

The following parameters were collected: (i) clinical parameters (past medical history, physical examination, electrocardiogram, arterial blood pressure, NYHA class, pharmacological therapy); (ii) echocardiographic parameters (ventricular chambers size, systolic and diastolic function, valve disease and severity, tricuspid annular plane systolic excursion [TAPSE]); (iii) laboratory parameters (blood cell count, creatinine, electrolytes, estimated glomerular filtration rate [eGFR], NT-pro BNP).

The study cohort was stratified according to HLM stage (HLM-1–HLM-4) which refers to increasing severity of disease, and according to SGLT2i status at the moment of hospital discharge. SGLT2is were administered according to HF guideline indications [1]. The difference in SGLT2i timing initiation was due to the 2023-focused update of HF guidelines [4]. The primary outcome was the composite of CV death or HF hospitalization (HFH) at 6 months follow-up.

In particular, the specific aims of the present study were: (i) to explore the prognostic role of HLM score, by examining the occurrence of the composite event of CV death and HFH at 6 months follow-up, according to SGLT2i status; (ii) to describe the clinical impact of SGLT2i therapy according to HLM severity stage, through the stage-specific absolute risk reduction (ARR) and number needed to treat (NNT), providing a clinically meaningful interpretation of treatment benefit; (iii) to evaluate the prognostic impact of SGLT2i initiation within the index hospitalization for HF.

Data were anonymously collected in a dedicated electronic case report form. The study was conducted according to the Helsinki Declaration. The study protocol was approved by the local Ethical Committee (rif. 7068 approved on 8 May 2023).

### 2.1. The HLM Score

The HLM score was based on the HLM classification, considering each H, L and M value as a numerical variable (Table 1) [3].

The linear combination of the coefficients (multiplied by 10 and then rounded to the nearest integer) obtained from the Cox PH model for the hazard of the composite and each single outcome was used. The resulting score was:

HLM score = 2H + 3L + 1M

The corresponding HLM stages were:

- HLM score 2–6: HLM-1
- HLM score 7–11: HLM-2
- HLM score 12–16: HLM-3
- HLM score 17–20: HLM-4

The risk stages stratified patients according to their risk and to guaranteed an adequate number of subjects in each class (at least 10% of the total).

### 2.2. Statistical Analysis

Continuous variables are presented as mean ± standard deviation or median (interquartile range) according to distribution, while categorical variables are expressed as counts and percentages. Between-group comparisons across HLM stages were performed using one-way ANOVA or Kruskal–Wallis tests for continuous variables and chi-square tests for categorical variables. Event-free survival was evaluated using Kaplan–Meier analysis, and differences between groups were assessed using the log-rank test. Survival curves were stratified by HLM stage and according to SGLT2i therapy. Stage-specific treatment effect was quantified by calculating ARR and NNT based on life-table estimates at 180 days. The association between clinical variables and the composite endpoint (CV death or HFH) was assessed using Cox proportional hazards regression analysis. Variables with clinical relevance and potential confounding effect were included in the multivariable model. The statistical analysis plan, including study outcomes, was predefined before data analysis. Investigators were not blinded to treatment status during the statistical analyses because of the prospective observational design of the study. To account for treatment selection bias, a propensity score for early SGLT2i therapy was derived using logistic regression, including baseline demographic and clinical covariates and incorporated into the Cox model. In particular, the covariates included in the propensity score were age, male gender, previous HFH, cardiovascular risk factors (arterial hypertension, T2DM, dyslipidemia), ischemic etiology, baseline HF triple therapy (MRAs + BB + ACEis/ARNIs/ARBs), baseline loop diuretic therapy, vericiguat therapy, baseline LVEF, eGFR and TAPSE. Hazard ratios (HRs) with 95% confidence intervals (CIs) were reported. The proportional hazards assumption was verified graphically using log-minus-log survival plots. For all tests, a *p*-value < 0.05 was considered statistically significant.

The statistical analysis was performed using SPSS version 27.0 for Mac (IBM Software, Inc., Armonk, NY, USA).

## 3. Results

A total of 711 consecutive patients were enrolled between November 2022 and August 2025. Mean age of the study population was 72.7 ± 11.8 years; 488 (68.6%) patients were male, 406 (57.1%) patients had ischemic HF etiology, and 252 (35.5%) patients had a prior HF hospitalization. Baseline characteristics of the overall cohort and stratified by HLM severity stage and according to SGLT2i status are reported in Table 2 and Table 3, respectively.

The distribution of guideline-directed HF therapies, loop diuretics, and vericiguat across HLM stages is shown in Table 4. Regarding SGLT2is, 264 (68%) patients were treated with dapagliflozin and 125 (32%) patients with empagliflozin. No significant differences in sex distribution between patients treated and untreated with SGLT2is were observed within any HLM stage (HLM-1: *p* = 0.283; HLM-2: *p* = 0.454; HLM-3: *p* = 0.725; HLM-4: *p* = 0.101).

During follow-up, the composite endpoint of CV death or HFH occurred in 82 patients (11.5%), with significant differences among HLM stages (*p* < 0.001). Significant differences among HLM stages were also observed for CV death (*p* < 0.001) and HFH (*p* = 0.04), whereas worsening HF episodes did not differ significantly among groups (Table 5).

Stage-stratified survival analysis showed consistent survival benefit associated with SGLT2i therapy across HLM stages, with numerically different ARR reflecting differences in baseline risk. In HLM-1, event-free survival was 97% in treated patients versus 90% in untreated patients (ARR = 7%; NNT = 14). In HLM-2, survival was 95% with SGLT2is versus 87% without treatment (ARR = 8%; NNT = 13). In HLM-3, survival was 84% versus 80% (ARR = 4%; NNT = 25). In HLM-4, survival was 79% versus 65% (ARR = 14%; NNT = 7) (Table 6).

**Figure 1:**
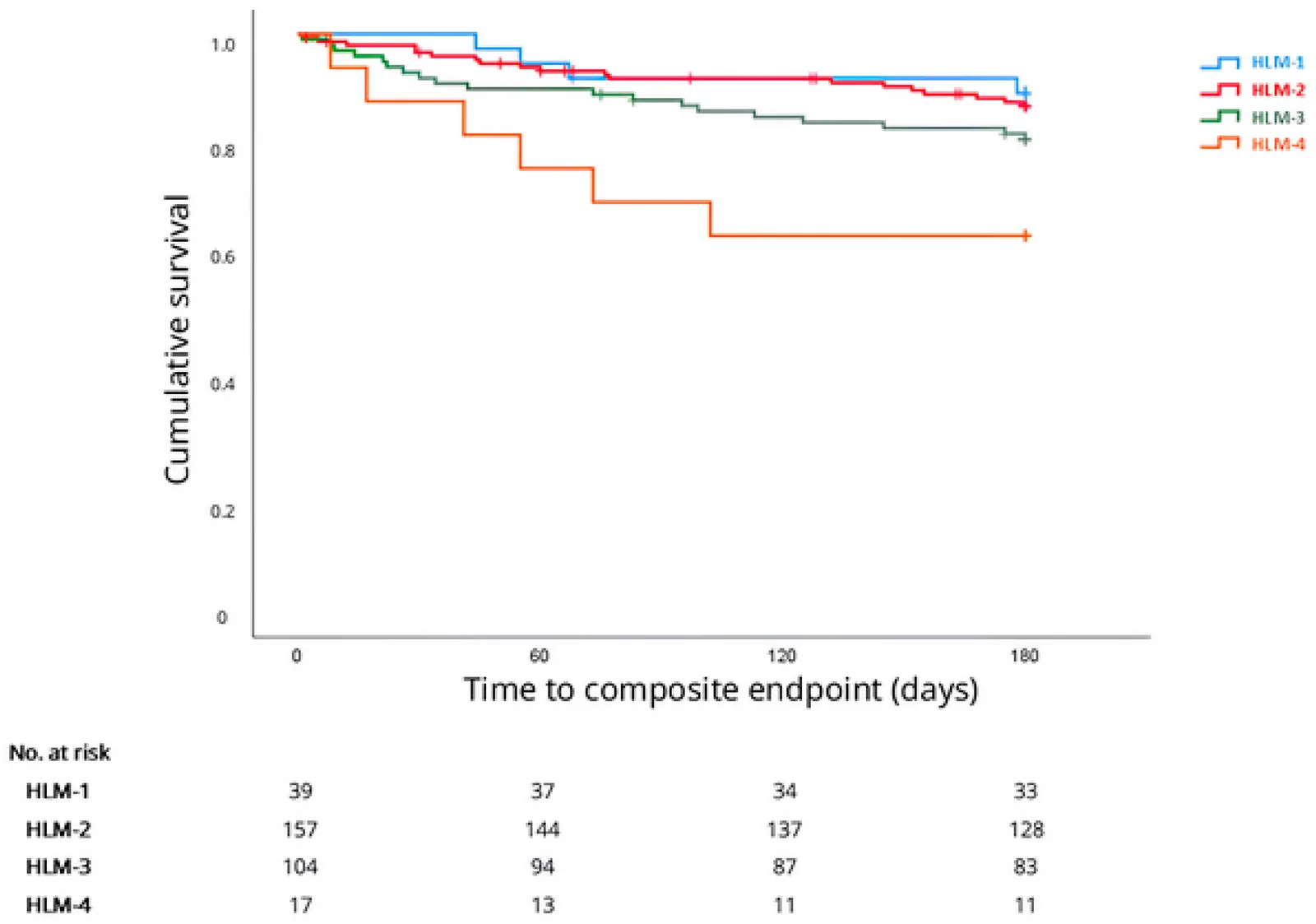
Kaplan–Meier survival analysis across HLM stages in untreated patients.

**Figure 2:**
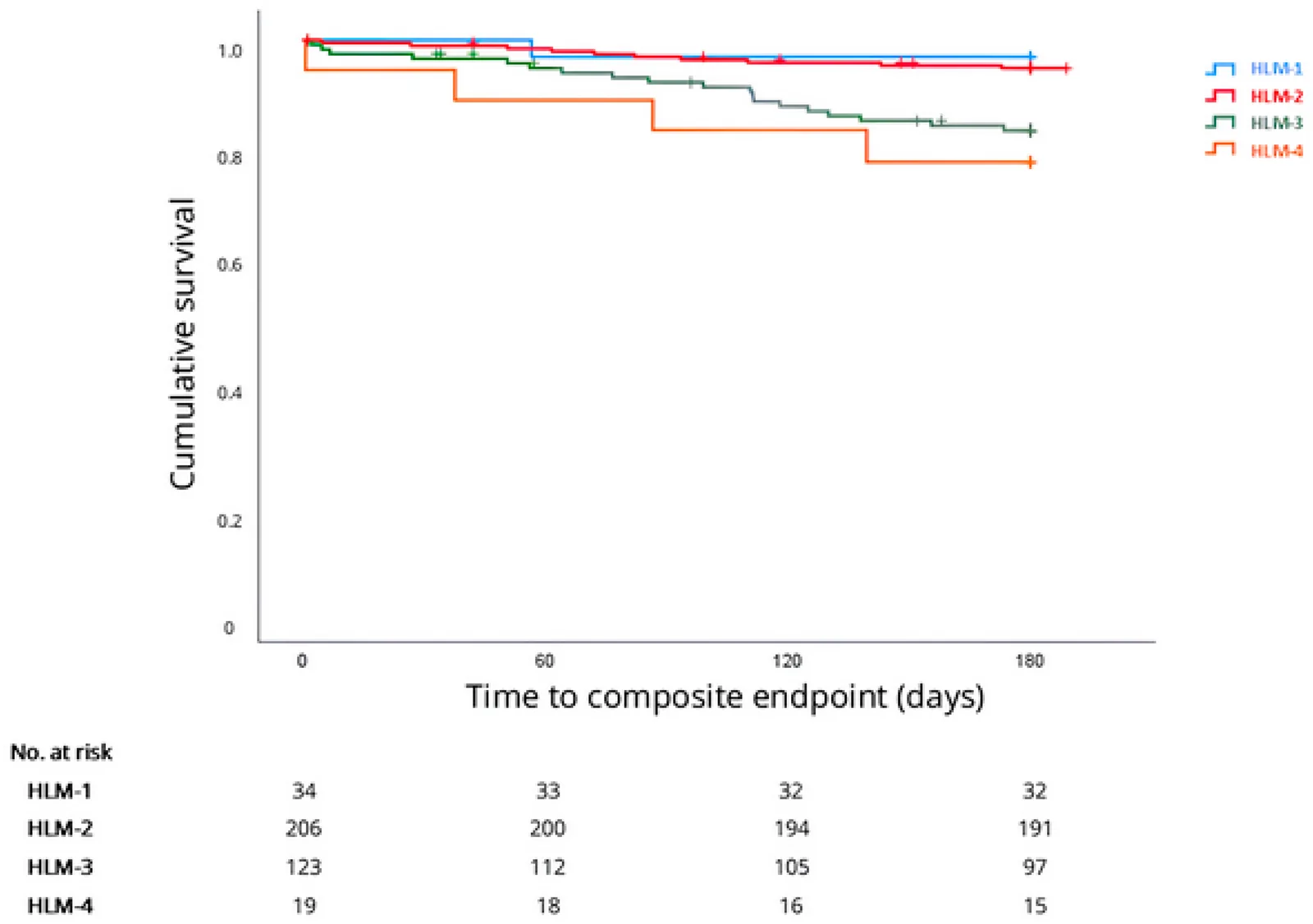
Kaplan–Meier survival analysis across HLM stages in patients receiving SGLT2i.

Kaplan–Meier analysis confirmed a stepwise reduction in event-free survival across HLM stages both in untreated patients (log-rank *p* = 0.03) and in patients receiving SGLT2is (log-rank *p* = 0.001 (Figure 1 and Figure 2).

After adjustment using a propensity score to account for major confounders, multivariable Cox regression analysis demonstrated that increasing HLM stage was independently associated with a higher risk of the composite outcome (HR = 1.40; 95% CI 1.02–1.90; *p* = 0.03), irrespective of SGLT2i therapy, while SGLT2i initiation within the index hospitalization due to HF was associated with a reduced risk of the composite endpoint (HR = 0.59; 95% CI 0.38–0.92; *p* = 0.02) across the HLM severity stages.

An additional Cox model including a SGLT2i treatment × HLM stage interaction term showed no significant interaction between HLM stage and SGLT2i therapy (HR 1.44, 95% CI 0.79–2.60; *p* = 0.23).

## 4. Discussion

In the present study, HF severity assessed through the HLM score was strongly associated with adverse outcomes, with a progressive increase in CV death and HFH across HLM stages, irrespective of SGLT2i status. Moreover, SGLT2i initiation within an index hospitalization due to HF was associated with improved clinical outcomes at 6 months follow-up, across the entire spectrum of HLM severity. This suggests that increasing multiorgan involvement in HF does not attenuate the observed treatment benefit of SGLT2is.

HF is a complex clinical syndrome characterized by structural and/or functional cardiac abnormalities leading to inadequate cardiac output and/or elevated intracardiac filling pressures [1]. However, growing evidence indicates that HF extends beyond a purely cardiocentric disorder and should be regarded as a systemic disease involving multiple organs and regulatory pathways [5]. HF is frequently accompanied by a substantial burden of non-cardiac comorbidities, including CKD, T2DM, chronic obstructive pulmonary disease, anemia, obesity, and metabolic syndrome, all of which significantly influence clinical outcomes and functional status [6,7].

The pathophysiology of HF reflects complex interactions among cardiac dysfunction, renal impairment, metabolic abnormalities, systemic inflammation, and neurohormonal activation [8]. Importantly, traditional classifications based on LVEF or NYHA functional class incompletely capture this biological complexity [2]. LVEF, although widely used for diagnostic and therapeutic stratification, shows important limitations in reproducibility and in reflecting disease severity, particularly at higher values [2,9]. Similarly, NYHA class demonstrates substantial overlap across objective functional and biomarker measures and poorly discriminates risk among patients with comparable clinical profiles [10].

For this reason, a more integrated and pathophysiological-oriented staging approach has been proposed to better reflect the systemic nature of HF. The HLM scoring system was conceived as a TNM-like staging system for HF, incorporating three domains: cardiac damage (H), pulmonary involvement (L) and peripheral multiorgan dysfunction (M) [11]. By explicitly integrating extracardiac organ involvement, the HLM model aims to provide a clinically applicable and prognostically meaningful framework aligned with the multisystemic nature of HF [3].

Evidence from prior studies supports the prognostic relevance of the HLM staging system. In a large multicenter prospective cohort, HLM demonstrated superior predictive accuracy for CV death and HFH compared to NYHA class, American college of cardiology (ACC)/American heart association (AHA) stages, and LVEF, underscoring the added value of pathophysiological staging over conventional classifications [3]. Similarly, in patients with ischemic HF, HLM staging system independently predicted CV death and HFH, confirming its prognostic consistency across etiologic subsets [12].

In our cohort, progressive HLM severity stages were independently associated with an increased risk of the composite endpoint, regardless of SGLT2i status (HR = 1.40; 95% CI 1.02–1.90; *p* = 0.03). These findings reinforce the concept that HF progression reflects a systemic process in which the extent of organ involvement parallels worsening prognosis, highlighting the clinical relevance of integrated pathophysiological staging.

Within this framework, the benefit, in terms of CV death and HFH reduction, of early SGLT2i therapy appeared consistent across the entire spectrum of HLM severity (HR = 0.59; 95% CI 0.38–0.92; *p* = 0.02). This stage-transversal pattern suggests that the therapeutic impact of SGLT2is extends across the continuum of HF severity, from early stages with limited extracardiac involvement to advanced conditions characterized by substantial pulmonary and peripheral organ dysfunction.

Several mechanisms may explain this effect. SGLT2is restore tubule–glomerular feedback by increasing sodium delivery to the macula densa, promoting afferent arteriolar vasoconstriction and reducing intraglomerular pressure, thereby mitigating hyperfiltration and slowing renal functional decline [13]. Beyond the renal hemodynamic, SGLT2 inhibition induces natriuresis and osmotic diuresis, contributing to plasma volume regulation and ventricular loading optimization [14]. At the myocardial level, SGLT2is are associated with anti-remodeling effects and improved cardiac energetics [15,16,17,18]. Emerging observational evidence also suggests a potential reduction in ventricular arrhythmic burden in high-risk HF with reduced ejection fraction (HFrEF) patients treated with SGLT2is [19]. The persistence of favorable outcome signals even in advanced HLM stages suggests that the benefits of SGLT2is are not confined to early ventricular remodeling but extend to systemic processes characterizing multiorgan HF progression. Conversely, the signal observed in earlier stages supports the rationale for timely initiation, potentially before the development of extensive multiorgan damage.

Recent studies further support the feasibility and clinical relevance of early SGLT2i initiation in HF [20,21,22,23,24,25,26]. In the DAPA ACT HF–TIMI 68 trial [24], in-hospital initiation of dapagliflozin in stabilized patients was feasible and associated with early signals of clinical benefit without excess safety concerns. Real-world evidence also indicates that SGLT2i therapy remains generally well tolerated even in advanced HFrEF, although improvements in biomarkers or functional parameters may be less pronounced in later disease stages [26].

This consideration is particularly relevant when interpreting outcome in higher HLM stages. As emphasized in contemporary discussions on optimal timing and patient selection, SGLT2i therapy should be individualized, balancing hemodynamic stability, renal profile, and overall clinical context rather than relying on disease stage alone [21,24].

Overall, these findings potentially support the concept that SGLT2is act through systemic mechanisms targeting interconnected pathophysiological pathways of HF. Evaluating treatment response within a multiorgan staging framework such as the HLM scoring system may therefore provide a complementary perspective for risk stratification and therapeutic decision-making in contemporary HF management.

## 5. Limitations

This study should be interpreted in light of several considerations. Despite being a multicenter study, the observational design reflects real-world clinical practice but may limit the generalizability of findings to different healthcare settings or patient populations. Nevertheless, the prospective enrolment of consecutive patients and the comprehensive clinical characterization of the cohort support the internal consistency of the analysis. Although treatment allocation was not randomized, multivariable adjustment and propensity score modeling were applied to account for major baseline differences. While the possibility of residual confounding cannot be entirely excluded, these methods were intended to minimize treatment selection bias and to approximate real-world clinical decision processes. The number of events in the most advanced HLM stages was relatively small, resulting in wider confidence intervals for stage-specific estimates. Given the limited sample size in the HLM-4 subgroup, conclusions regarding advanced HF should be interpreted with caution, and the results should be considered exploratory. Studies with larger and more diverse populations and more detailed longitudinal assessment may help to refine these observations. Lastly, the relatively short follow-up period of 6 months limits the ability to draw conclusions regarding the longer-term course of the disease. Therefore, the present findings should be interpreted as reflecting short-term outcomes and warrant confirmation in studies with longer follow-up.

## 6. Conclusions

In this real-world cohort of patients with HF, the HLM staging system confirmed its value as an integrated and clinically meaningful indicator of disease severity, reflecting the systemic and multiorgan nature of the syndrome. Increasing HLM stage was independently associated with a higher risk of adverse outcomes, supporting the relevance of pathophysiological staging beyond conventional cardiocentric classifications.

Early SGLT2i therapy within an index hospitalization due to HF was associated with improved clinical outcomes across the entire spectrum of HLM severity, with no evidence of attenuation of benefit in more advanced stages of multiorgan involvement. These findings may support the concept that a systemic therapeutic approach may remain effective across different levels of disease complexity [27,28,29,30]. The observed alignment between a multiorgan staging model and a therapy exerting multisystem effects reinforces the importance of integrated approaches to both risk stratification and treatment in contemporary HF management [28,29].

From a clinical perspective, these findings potentially suggest that HLM staging may provide additional support in risk stratification and may help contextualize the potential benefit of SGLT2i therapy across different degrees of HF severity. Furthermore, the consistent benefit of SGLT2is observed across stages may support their early initiation [27], as well as their use in patients with more advanced disease, although larger studies are needed to better define stage-specific effects.

## Figures and Tables

**Table 1:** HLM classification.

Heart (H)	Lungs (L)	Malfunction of Other Organs (M)
**H1:** Diastolic dysfunction and/or presence of structural cardiac damage * in absence of LV systolic dysfunction (LVEF ≥ 50%) **H2:** LV systolic (LVEF < 50%) or diastolic dysfunction with structural damage without LV dilation **H3:** LV dilation, structural cardiac damage with systolic (LVEF < 50%) or diastolic dysfunction, or right ventricular systolic dysfunction (TAPSE < 17 mm)	**L0:** Absence of any lung involvement	**M0:** Absence of malfunction of other organs **
	**L1:** Hemodynamic lung involvement, assessed by CXR, and/or sPAP ≥ 35 mmHg at rest, assessed by TTE, with absence of clinical signs of lung congestion	**M1:** Presence of a single end-organ damage (except heart and lungs)
	**L2:** Clinical signs and symptoms of lung congestion assessed by physical examination (crepitation, raised jugular venous pressure, orthopnea, dyspnea, necessity of supplemental oxygen) and increase in left ventricular filling pressure, assessed by echocardiographic evaluation and, if feasible, by right heart catheterization	**M2:** Presence of two distinct end-organ damages (except heart and lungs)
**H4:** Biventricular systolic dysfunction (LVEF < 50% and TAPSE < 17 mm)	**L3:** “Cardiac lung” defined by arterialization of pulmonary vasculature, with post-capillary hypertension (type II) and necessity of supplemental oxygen at discharge, despite use of congestion-relief therapy and absence of congestion.	**M3:** Presence of ≥3 end-organ damage (except heart and lungs)

LV: left ventricle; LVEF: left ventricle ejection fraction; TAPSE: tricuspid annular plane systolic excursion; CHF: congestive heart failure; CXR: chest x-ray; sPAP: systolic pulmonary arterial pressure; TTE: transthoracic echocardiography; GFR; glomerular filtration rate; AST: aspartate aminotransferase, ALT: alanine aminotransferase; BMI: body mass index; CT: computed tomography; MRI: magnetic resonance imaging. * Structural damage is defined by at least one of the following: abnormal wall motion, left ventricular hypertrophy, moderate to severe left-sided valvular disease. ** Malfunction of other organs is defined as: GFR < 60 mL/min regarding kidney dysfunction; elevation at least twice as high as normal for at least one parameter among AST/ALT/total bilirubin/gamma–glutamyl transferase/alkaline phosphatase regarding liver dysfunction; hemoglobin < 13 g/dL for men and <12 g/dL for women, regarding anemia; transferrin saturation < 20% with serum ferritin between 100 and 299 ng/mL or serum ferritin < 100 ng/mL alone, regarding iron deficiency; more than 5% edema-free body weight loss during the previous year or less, regarding HF-related cachexia; the Beck Depression Inventory and Cardiac Depression Scale were used to assess HF-related depression and anxiety disorders and CT/MRI to exclude ischemic or hemorrhagic stroke, regarding central nervous system involvement.

**Table 2:** Baseline features of study cohort according to HLM severity stage.

Variables	Overall Population (*n* = 711)	HLM-1 (*n* = 74)	HLM-2 (*n* = 368)	HLM-3 (*n* = 232)	HLM-4 (*n* = 37)	*p*-Value
Age, years (±SD)	72.7 (11.8)	66.8 (12)	73.6 (11)	73.2 (12)	72 (14)	<0.001
Male gender, *n* (%)	488 (68.6)	55 (74.3)	261 (71)	149 (64.2)	23 (62.2)	0.033
Ischemic etiology, *n* (%)	406 (57.1)	42 (57)	219 (59.5)	123 (53)	22 (59.5)	N.S.
Previous HFH, *n* (%)	252 (35.5)	19 (26)	121 (33)	96 (41.4)	16 (43.2)	0.005
Arterial hypertension, *n* (%)	583 (82)	61 (82.4)	314 (85.3)	178 (76.7)	30 (81)	N.S.
Diabetes mellitus, *n* (%)	248 (35)	18 (24.3)	132 (36)	86 (37)	12 (32.4)	N.S.
Dyslipidemia, *n* (%)	502 (70.6)	59 (79.7)	280 (76)	142 (61.2)	21 (56.8)	<0.001
Smoking habit, *n* (%)	273 (38.4)	32 (43.2)	141 (38.3)	81 (35)	19 (51.4)	N.S.
COPD, *n* (%)	143 (20)	5 (6.8)	53 (14.4)	75 (32.2)	10 (27)	<0.001
CKD, *n* (%)	349 (49)	22 (29.7)	180 (49)	122 (52.6)	25 (67.6)	<0.001
Anemia, *n* (%)	267 (37.6)	19 (25.7)	117 (32)	106 (45.7)	25 (67.6)	<0.001
Atrial fibrillation, *n* (%)	248 (35)	20 (27)	116 (31.5)	94 (40.5)	18 (48.6)	0.002
Hemoglobin, g/dL (±SD)	12.9 (2.2)	13.6 (1.9)	13.2 (1.9)	12.6 (2.4)	11.5 (2.7)	<0.001
eGFR, mL/min (±SD)	60.3 (24.6)	69.7 (23.8)	61 (24.2)	58.3 (25)	49.5 (20)	<0.001
K+, mmol/(±SD)	4.8 (0.7)	4.5 (0.5)	5 (1)	4.3 (0.7)	4 (0.6)	N.S.
Na, mmol/L (±SD)	139 (9.2)	140 (4.8)	139 (3.8)	139 (4)	137 (5)	N.S.
LVEF, % (±SD)	39 (12)	42.6 (12)	41.2 (12)	36 (13)	28 (11)	<0.001
E/e’ ratio (±SD)	9 (2)	6 (1.8)	9 (1.7)	12 (1.7)	15 (1.8)	<0.001
TAPSE, mm (±SD)	19.2 (4.1)	20 (4.4)	20 (3.8)	18 (4)	16 (4)	<0.001
LVEDD, mm (±SD)	55 (8.8)	55 (8)	55 (8)	57 (9)	60 (9)	<0.001
HFrEF, *n* (%)	387 (54)	29 (39)	169 (46)	157 (68)	32 (86)	<0.001
HFmrEF, *n* (%)	134 (19)	17 (23)	87 (24)	26 (11)	4 (11)	<0.001
HFpEF, *n* (%)	190 (27)	28 (38)	112 (30)	49 (21)	1 (3)	<0.001

CKD: chronic kidney disease; COPD: chronic obstructive pulmonary disease; eGFR: estimated glomerular filtration rate; HFrEF: heart failure with reduced ejection fraction; HFmrEF: heart failure with mildly reduced ejection fraction; HFpEF: heart failure with preserved ejection fraction; HFH: heart failure hospitalization; IVS: interventricular septum; K: potassium; LVEF: left ventricular ejection fraction; Na: sodium; PW: posterior wall; SD: standard deviation; TAPSE: tricuspid annular plane systolic excursion; LVEDD: left ventricular end diastolic diameter; N.S.: not significant (*p* ≥ 0.05).

**Table 3:** Baseline features of study cohort according to SGLT2i status.

Variables	Overall Population (*n* = 711)	SGLT2i (*n* = 389)	No SGLT2i (*n* = 322)	*p*-Value
Age, years (±SD)	72.7 (11.8)	73 (11.6)	72.4 (12)	N.S.
Male gender, *n* (%)	488 (68.6)	263 (67.6)	225 (70)	N.S.
Ischemic etiology, *n* (%)	406 (57.1)	213 (54.8)	193 (60)	N.S.
Previous HFH, *n* (%)	252 (35.5)	137 (35.2)	115 (35.7)	N.S.
Arterial hypertension, *n* (%)	583 (82)	319 (82)	264 (82)	N.S.
Diabetes mellitus, *n* (%)	248 (35)	139 (35.7)	109 (34)	N.S.
Dyslipidemia, *n* (%)	502 (70.6)	292 (75.1)	210 (65)	0.004
Smoking habit, *n* (%)	273 (38.4)	152 (39)	121 (37.6)	N.S.
COPD, *n* (%)	143 (20)	71 (18.3)	72 (22.4)	N.S.
CKD, *n* (%)	349 (49)	190 (49)	159 (49.4)	N.S.
Anemia, *n* (%)	267 (37.6)	130 (33.4)	137 (42.5)	0.012
Atrial fibrillation, *n* (%)	248 (35)	139 (35.7)	109 (34)	N.S.
Hemoglobin, g/dL (±SD)	12.9 (2.2)	13 (2.2)	12.8 (2.1)	N.S.
eGFR, mL/min (±SD)	60.3 (24.6)	60 (23)	60 (26)	N.S.
K+, mmol/(±SD)	4.8 (0.7)	4.6 (0.7)	5 (0.8)	N.S.
Na, mmol/L (±SD)	139 (9.2)	139 (10)	139 (8)	N.S.
LVEF, % (±SD)	39 (12)	39.7 (13)	38 (12.3)	N.S.
E/e’ ratio (±SD)	9 (2)	9 (2.1)	10 (1.8)	N.S.
TAPSE, mm (±SD)	19.2 (4.1)	19 (4)	19 (4)	N.S.
LVEDD, mm (±SD)	55 (8.8)	55.6 (9)	55.6 (8)	N.S.
IVS, mm (±SD)	11 (2.2)	11 (2)	11 (2)	N.S.
PW, mm (±SD)	9.6 (1.5)	10 (1)	10 (1.5)	N.S.
HFrEF, *n* (%)	387 (54)	199 (51)	188 (58)	N.S.
HFmrEF, *n* (%)	134 (19)	73 (19)	61 (19)	N.S.
HFpEF, *n* (%)	190 (27)	117 (30)	73 (23)	0.030

CKD: chronic kidney disease; COPD: chronic obstructive pulmonary disease; eGFR: estimated glomerular filtration rate; HFrEF: heart failure with reduced ejection fraction; HFmrEF: heart failure with mildly reduced ejection fraction; HFpEF: heart failure with preserved ejection fraction; HFH: heart failure hospitalization; IVS: interventricular septum; K: potassium; LVEDD: left ventricular end diastolic diameter; LVEF: left ventricular ejection fraction; Na: sodium; PW: posterior wall; SD: standard deviation; TAPSE: tricuspid annular plane systolic excursion; N.S.: not significant (*p* ≥ 0.05).

**Table 4:** Distribution of guideline-directed HF therapies, loop diuretics and vericiguat across HLM stages.

Variables	Overall Population (*n* = 711)	HLM-1 (*n* = 74)	HLM-2 (*n* = 368)	HLM-3 (*n* = 232)	HLM-4 (*n* = 37)	*p*-Value
ACEis, *n* (%)	171 (24)	23 (31)	101 (27.5)	43 (18.5)	4 (11)	0.001
ARBs, *n* (%)	57 (8)	7 (9.5)	31 (8.4)	16 (7)	3 (8)	N.S.
ARNIs, *n* (%)	337 (47.4)	37 (50)	161 (43.8)	113 (48.7)	26 (70)	N.S.
BBs, *n* (%)	659 (93)	69 (93)	341 (92.7)	215 (92.7)	34 (92)	N.S.
MRAs, *n* (%)	443 (62.4)	45 (61)	216 (58.7)	156 (67.2)	26 (70)	N.S.
SGLT2is, *n* (%)	389 (55)	35 (47)	209 (56.8)	125 (54)	20 (54)	N.S.
Furosemide, *n* (%)	410 (57.7)	17 (23)	195 (53)	165 (71.1)	33 (89)	<0.001
Vericiguat, *n* (%)	71 (10)	25 (33.8)	31 (8.4)	13 (5.6)	2 (5.4)	<0.001

ACEis: angiotensin-converting enzyme inhibitors; ARBs: angiotensin II receptor blockers; ARNIs: angiotensin receptor–neprilysin inhibitors; BBs: beta-blockers; MRAs: mineralocorticoid receptor antagonists; SGLT2is: sodium–glucose cotransporter 2 inhibitors; N.S.: not significant (*p* ≥ 0.05).

**Table 5:** Distribution of the adverse events across the different HLM stages.

Variables	Overall Population (*n* = 711)	HLM-1 (*n* = 74)	HLM-2 (*n* = 368)	HLM-3 (*n* = 232)	HLM-4 (*n* = 37)	*p*-Value
Composite endpoint, *n* (%)	82 (11.5)	7 (9.5)	27 (7.3)	38 (16.4)	10 (27)	<0.001
CV death, *n* (%)	30 (4.2)	1 (1.4)	5 (1.4)	18 (7.8)	6 (16.2)	<0.001
HFH, *n* (%)	59 (8.3)	6 (8.1)	24 (6.5)	24 (10.3)	5 (13.5)	0.04
WHF, *n* (%)	82 (11.5)	7 (9.5)	37 (10.1)	31 (13.4)	7 (19)	N.S.

CV: cardiovascular; HFH: heart failure hospitalization; WHF: worsening heart failure; N.S.: not significant (*p* ≥ 0.05).

**Table 6:** Clinical benefit of SGLT2i therapy variation according to HLM severity stages. ARR, NNT and 95% CI were derived from life-table estimates at 180 days.

Variables	Survival SGLT2i (SE)	Survival No SGLT2i (SE)	Risk SGLT2i	Risk No SGLT2i	ARR (95% CI)	NNT
HLM-1	0.97 (0.03)	0.90 (0.05)	0.03	0.10	7% (−4.4; 18.4)	14
HLM-2	0.95 (0.02)	0.87 (0.03)	0.05	0.13	8% (0.9; 15.1)	13
HLM-3	0.84 (0.03)	0.80 (0.04)	0.16	0.20	4% (−5.8; 13.8)	25
HLM-4	0.79 (0.12)	0.65 (0.12)	0.21	0.35	14% (−19.3; 47.3)	7

ARR: absolute risk reduction; CI: confidence interval; NNT: number needed to treat; SGLT2is: sodium–glucose cotransporter 2 inhibitors; SE: standard error.

## Data Availability

The data presented in this study are available on request from the corresponding author.

## Abbreviations

The following abbreviations are used in this manuscript:

ACC	American College Of Cardiology
ACEi	Angiotensin-converting enzyme inhibitor
AHA	American Heart Association
ALT	Alanine aminotransferase
ARNI	Angiotensin receptor–neprilysin inhibitor
ARR	Absolute risk reduction
AST	Aspartate aminotransferase
BB	Beta-blocker
BMI	Body mass index
CHF	Congestive heart failure
CI	Confidence interval
CKD	Chronic kidney disease
COPD	Chronic obstructive pulmonary disease
CT	Computed tomography
CV	Cardiovascular
CXR	Chest x-rays
eGFR	Estimated glomerular filtration rate
ESC	European Society of Cardiology
HF	Heart failure
HFH	Heart failure hospitalization
HFrEF	Heart failure with reduced ejection fraction
HR	Hazard ratio
IVS	Interventricular septum
K	Potassium
LV	Left ventricle
LVEDD	Left ventricular end diastolic diameter
LVEF	Left ventricular ejection fraction
MRI	Magnetic resonance imaging
MRA	Mineralocorticoid receptor antagonists
Na	Sodium
NNT	Number needed to treat
NYHA	New York Heart Association
PW	Posterior wall
SD	Standard deviation
SE	Standard error
SGLT2i	Sodium–glucose cotransporter 2 inhibitor
sPAP	Systolic pulmonary arterial pressure
T2DM	Diabetes mellitus type II
TAPSE	Tricuspid annular plane systolic excursion
TTE	Transthoracic echocardiography
